# Genetic Landscape of Slovenians: Past Admixture and Natural Selection Pattern

**DOI:** 10.3389/fgene.2018.00551

**Published:** 2018-11-19

**Authors:** Pierpaolo Maisano Delser, Metka Ravnik-Glavač, Paolo Gasparini, Damjan Glavač, Massimo Mezzavilla

**Affiliations:** ^1^Smurfit Institute of Genetics, Trinity College Dublin, Dublin, Ireland; ^2^Department of Zoology, University of Cambridge, Cambridge, United Kingdom; ^3^Department of Molecular Genetics, Institute of Pathology, Faculty of Medicine, University of Ljubljana, Ljubljana, Slovenia; ^4^Institute of Biochemistry, Faculty of Medicine, University of Ljubljana, Ljubljana, Slovenia; ^5^Institute for Maternal and Child Health, IRCCS “Burlo Garofolo”, Trieste, Italy; ^6^Department of Medical, Surgical and Health Sciences, University of Trieste, Trieste, Italy

**Keywords:** Slovenia, human, single nucleotide polymorphism, demographic histories, selection, admixture

## Abstract

The Slovenian territory played a crucial role in the past serving as gateway for several human migrations. Previous studies used Slovenians as a source population to interpret different demographic events happened in Europe but not much is known about the genetic background and the demographic history of this population. Here, we analyzed genome-wide data from 96 individuals to shed light on the genetic role and history of the Slovenian population. Y chromosome diversity splits into two major haplogroups R1b and R1a with the latter suggesting a genetic contribution from the steppe. Slovenian individuals are more closely related to Northern and Eastern European populations than Southern European populations even though they are geographically closer. This pattern is confirmed by an admixture and clustering analysis. We also identified a single stream of admixture events between the Slovenians with Sardinians and Russians around ∼2630 BCE (2149-3112). Using ancient samples, we found a significant admixture in Slovenians using Yamnaya and the early Neolithic Hungarians as sources, dated around ∼1762 BCE (1099-2426) suggesting a strong contribution from the steppe to the foundation of the observed modern genetic diversity. Finally, we looked for signals of selection in candidate variants and we found significant hits in *HERC2* and *FADS* responsible for blue eye color and synthesis of long-chain unsaturated fatty acids, respectively, when Slovenians were compared to Southern Europeans. While the comparison was done with Eastern Europeans, we identified significant signals in *PKD2L1* and *IL6R* which are genes associated with taste and coronary artery disease, respectively.

## Introduction

The Slovenian territory is geographically located between the Alps, the Adriatic Sea and the Pannonian basin and as such it could have been used as a gateway for different populations over several periods of time. However, the presence of geographical and possibly cultural barriers could have led to a more puzzling role for this region. The territory of modern day Slovenia was settled during the 6th and 7th centuries AD by different Slavic tribes from at least two different directions: one from the north and one from the east (Grafenauer, 1950). The origin of the present-day Slovenian population and their language is still debate between different theories regarding the Slavic migration and settlement (Grafenauer, 1950; Žužek, 2007). Indeed, there are several hypotheses about a South Slavic influence or a West Slavic origin followed by a South Slavic contribution (Grafenauer, 1950; Bezljaj, 1967; Žužek, 2007). However, this background gives us only little clues regarding the genetic features of the founder pool.

Previous genetic studies were only based on Y chromosome variation (Zupan et al., 2013) showing strong affinity of the Slovenians with West Slavic populations. However, a fine characterization of the Slovenia population using genome wide data is missing even though this population was used in previous studies (Esko et al., 2013). Therefore, a gap is left on what we know about the history and the role of the Slovenian population in the past. Furthermore, the availability of ancient genomes during the last years empowered our ability to better understand the history of modern and past populations (Haber et al., 2016); in particular several papers revealed a significant contribution of Yamnaya population to Europeans (Haak et al., 2015). According to the authors, the steppe ancestry persisted in all sampled central Europeans until at least 3,000 years ago, and it is equally distributed in present-day Europeans, providing also evidences for a steppe-origin of the Indo-European languages (Haak et al., 2015).

All these elements highlight the necessity of a broader and more comprehensive genetic study on Slovenian population. Outcomes of this kind of study could be useful to several disciplines and not only for population genetics and linguistics. For example, in the context of genetic epidemiology, describing the genetic landscape of this population would be beneficial to understand the genetic background of disease-related loci and their distribution; in fact Slovenians show evidence of familial hypercholesterolemia (3.1%) (Catapano et al., 2016), a disease characterized by increased risk of coronary heart disease. A recent report also showed that more than 10% of women in Slovenia are affected by heart or circulation problems (Townsend et al., 2016).

Therefore, our aims are to provide (i) a detailed characterization of the genetic structure of Slovenians in a broader European context using both Y chromosome and autosomal data, (ii) a description of the past and present admixture pattern, and (iii) a survey of variants putatively under selection and associated with different traits/diseases. For these purposes, we used genotype data from 96 Slovenian individuals and we analyzed them together with previously published modern and ancient samples.

## Materials and Methods

### Samples Data and QC

**FIGURE 1:**
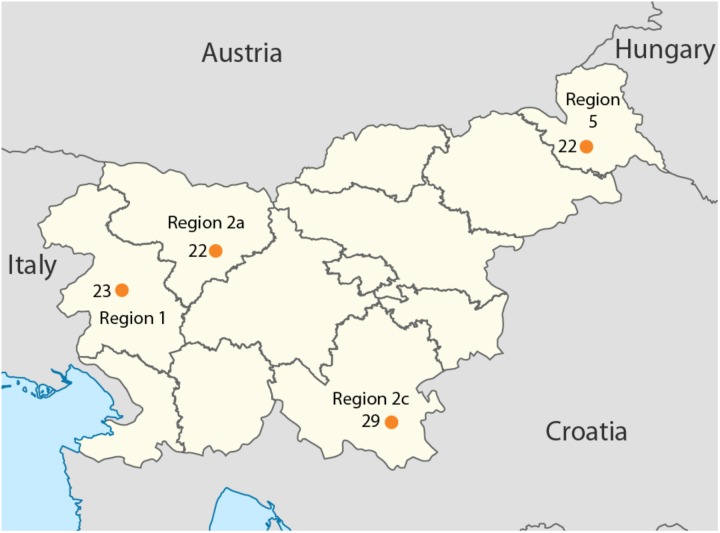
Geographical location of the samples included in this study. Sample size is reported next to each sampling location together with the region ID. Map has been modified from https://nl.m.wikipedia.org/wiki/Bestand:Slovenia,_administrative_divisions_-_de_(statistical_regions).svg.

Overall, 96 samples ranging from Slovenian littoral to Lower Styria were genotyped for 713,599 markers using the OmniExpress 24-V1 BeadChips (Figure 1), genetic data were obtained from Esko et al. (2013). After removing related individuals, 92 samples were left. The Slovenian dataset has been subsequently merged with the Human Origin dataset (Lazaridis et al., 2016) for a total of 2163 individuals. Only population with a minimum samples size of 10 individuals were retained and related individuals were discarded (PI_HAT cut-off of 0.125 in PLINK Purcell et al., 2007). The final dataset includes 1116 individuals grouped in 55 populations distributed worldwide (Slovenia_HO dataset). Finally, a subset including only European populations was also created and it groups 443 individuals from 20 populations (Slovenian_HO_EU). We also generated a dataset containing ancient genomes form Haak et al. (2015) (Slovenian_AG). Additional details can be found in Supplementary Table 1.

### Y Chromosome Analyses

First, 52 male samples were extracted from the dataset and Y chromosome haplogroups were assigned using AMY-tree v2.0 software (Van Geystelen et al., 2013). Input files were created by converting PED and FAM files into a vcf using PGDSpider v2.1.1.1 (Lischer and Excoffier, 2012) and then from a vcf into AMY-tree input files with R scripts v3.2.3 (R Core Team, 2015). Results were then combined together using in-house R scripts v3.2.3 (R Core Team, 2015).

### Population Structure

Principal Component Analysis (PCA) was performed using the option –pca in PLINK v1.9 (Purcell et al., 2007). Presence of genetic clusters was assessed using Gaussian mixture modeling implemented in the library “Mclust” v1.0 (Fraley et al., 2016) which uses several clustering models based on different characteristics of the geometric distribution (volume, shape, and orientation). Cluster plots were generated in R environment using the library “adegenet” v1.4-2 (Jombart and Ahmed, 2011). Pairwise Fst was calculated using the program 4P (Benazzo et al., 2015), and a UPGMA tree was then built with the R package “phangorn” (Schliep, 2011) including all populations listed in Supplementary Table 1 without any filter for sample size. Runs of homozygosity (ROH) were calculated using PLINK v1.9 (Purcell et al., 2007) on the dataset Slovenian_HO_EU. ROH hotspots were estimated using the command –homozyg-group implemented in PLINK using default parameters and all Slovenians samples. Genes contained in the ROH hotpots shared by at least 10 individuals were analyzed with BioMart (Zerbino et al., 2018). Genes found associated to trait in GWAS catalog were also reported.

### Admixture Pattern

We inferred the ancestral structure using ADMIXTURE v1.30 (Alexander et al., 2009), using maximum likelihood for components (*K*) from *K* = 2 to 12. Cross validation error was also performed. Gene flow and time of admixture between Slovenian and modern populations in the Slovenian_HO_EU dataset was investigated using ALDER v.1.30 (Loh et al., 2013) and MALDER (Pickrell et al., 2014). These two approaches exploit the decay of linkage disequilibrium as a function of genetic distance. Slovenian population was always used as a target of admixture between two sources. All possible pairs of populations with a sample size greater than 10 were used as sources. Ancient gene flow was assessed using the f3 statistic implemented in ADMIXTOOLS (Patterson et al., 2012) using the dataset containing ancient genomes. Only significant results with *Z*-score <= -4 were retained and ALDER was then performed in the ancient reference population with sample size >= 5. The outgroup f3 statistic was estimated in the form (Modern; Ancient, Yoruba) for modern populations with sample size greater than 10.

### Natural Selection

Analyses looking for signals of selection were performed using PCAdapt implemented in the R package PCAdapt (Luu et al., 2017). A 2-population system was applied comparing Slovenian population with one South European reference, one East European, one North European, and one West European. Markers with False discovery rate (FDR) < 0.1 were analyzed and SNPs reported in GWAS catalog associated with a trait with *p*-value < 1e-8 were then selected. Furthermore, markers reported in Mathieson et al. (2015) were also analyzed as potential candidates in Europeans.

## Results

### Genetic Context of the Slovenian Population

First, Y chromosome genetic diversity was assessed. A total of 52 Y chromosomes were analyzed for 195 SNPs. The majority of individuals (25, 48.1%) belong to the haplogroup R1a1a1a (R-M417) while the second major haplogroup is represented by R1b (R-M343) including 15 individuals (28.8%). Twelve samples are assigned to haplogroup I (I M170): five and two samples belong to haplogroup I2a (I L460) and I1 (I M253), respectively, while the remaining five samples did not have enough information to be further assigned. We then performed principal component analysis on autosomal data to further investigate the presence of structure within the Slovenian population. We did not find any clusters even when samples were highlighted by the region of origin (Supplementary Figure 1).

**FIGURE 2:**
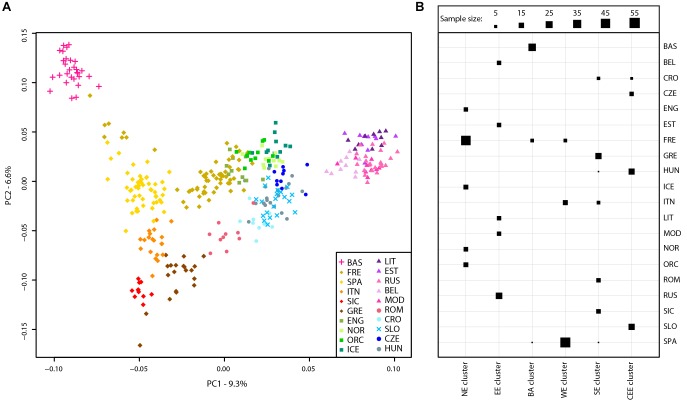
Population structure of Slovenian samples. **(A)** PCA of Slovenian samples with European populations (Slovenian_HO_EU dataset). For details regarding the populations included, see Supplementary Table 1. **(B)** Mclust clustering (Fraley et al., 2016) of European samples based on the first eight principal component of the PCA. The number of clusters is *k* = 6 as suggested by multiple clustering models, see Supplementary Figure 3. Horizontal lines represent populations while vertical lines represent the six clusters. The size of each square is proportional to the number of individuals assigned to each cluster as shown in the figure legend. For population acronyms see Supplementary Table 1. NE, Northern European cluster; EE, Eastern European cluster; BA, Basque cluster; WE, Western European cluster; SE, Southern-European cluster; CEE, Central-Eastern European cluster.

A principal component analysis was then performed on the Slovenian_HO dataset including 127,385 SNPs. The first two PCs explained ∼63% of the variance and PC1 divides the African samples while PC2 highlights the Asian cluster (Supplementary Figure 2). PC3 separates the Caucasian and Middle Eastern populations but all Slovenian individuals group within the European cluster. To further investigate the role of the Slovenia genetic diversity within the European scenario, a principal component analysis was also carried out on the Slovenia_HO_EU dataset. Considering the unbalanced sample size of the Slovenian population compared to the other populations included in the dataset, a subset of 20 Slovenian individuals randomly sampled was used. In order to verify that the 20 Slovenian individuals did not show any bias (i.e., population structure), this approach has been replicated 10 times and PCAs showed the same pattern. One of the replicates of the PCAs is shown in Figure 2A. The first two PCs explained ∼16% of the variance with Slovenian samples grouping together with the Croatians, Hungarians and close to the Czechs. PC1 represents a west-to-east cline with the Basque at one extreme and the “Eastern-European” cluster made by Lithuanians, Estonians, Russians, Belarusians, and Mordovians at the opposite side. The second principal component highlights a north-to-south cline with English, Norwegians, Orcadians, and Icelanders at the top and Sicilians and Greeks at the bottom. PC3 explains ∼5.5% of the variance and emphasizes this cline by separating more clearly the French and the English from the Spanish and the other Northern-European populations, respectively.

The presence of genetic clusters within the Slovenia_HO_EU dataset has been further investigated using “Mclust” which suggests *k* = 6 as the most likely number of clusters (Figure 2B and Supplementary Figure 3). All Slovenian samples group together with Hungarians, Czechs, and some Croatians (“Central-Eastern European” cluster) as also suggested by the PCA. All Basque individuals with few French and Spanish cluster together (“Basque” cluster) while a “Northern-European” cluster is made of the majority of French, English, Icelanders, Norwegians, and Orcadians. Five populations contributed to the “Eastern-European” cluster including Belarusians, Estonians, Lithuanians, Mordovians, and Russians. Western and South Europe is split into two cluster: the first (“Western European” cluster) includes all Spanish individuals, few French, and some Italians (North Italy) while the second (“Southern-European” cluster) groups Sicilians, Greeks, some Croatians, Romanians, and some Italians (North Italy).

The relationships between populations were also assessed by computing a pairwise Fst matrix. Analysis of the UPGMA tree based on the Fst matrix shows all Slovenian individuals clustering together with Hungarians, Czechs, Croatians, Ukrainians, and Belarusians (Supplementary Figure 4).

Pattern of runs of homozygosity computed on the Slovenian population does not differ significantly from Hungarians, Czechs, Croatians (Mann Whitney *p*-value > 0.09), albeit showing a slightly higher amount of segments and total homozygosity (Supplementary Table 2). We investigated the ROH hotspot pattern and we found total of 13 regions in homozygous state shared by more than 10 individuals, the average sizes of regions is 435 KB. The regions range from 131 Kb to 988 Kb where we found a total of 159 genes, 14 of them are present in GWAS catalog with a marker associated with a reported *p*-value <= 1e-8. These genes are mainly involved in blood metabolites levels such as SLC7A6, RAB3GAP1, ADK, lipid traits (PLA2G15 and TP53BP1), and blood pressure (ACAD10 and HECTD4) (Supplementary Tables 3, 4).

### Admixture Pattern and Migration

**FIGURE 3:**
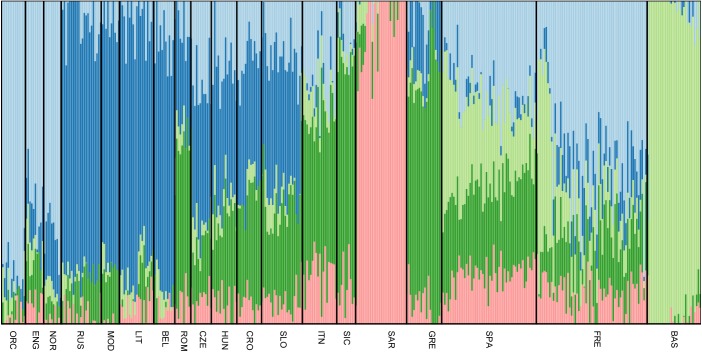
Unsupervised admixture analysis of Slovenians. Results for *K* = 5 are showed as it represents the lowest cross-validation error. Slovenian samples show an admixture pattern similar to the neighboring populations such as Croatians and Hungarians. The major ancestral components are: the blue one which is shared with Lithuanians and Russians, followed by the dark green one that is mostly present in Greek samples and the light blue which characterizes Orcadians and English. For population acronyms see Supplementary Table 1.

All Slovenian individuals share common pattern of genetic ancestry, as revealed by ADMIXTURE analysis. The three major ancestry components are the North East and North West European ones (light blue and dark blue, respectively, Figure 3), followed by a South European one (dark green, Figure 3). Contribution from the Sardinians and Basque are present in negligible amount. The admixture pattern of Slovenians mimics the one suggested by the neighboring Eastern European populations, but it is different from the pattern suggested by North Italian populations even though they are geographically close. (Figure 3).

**FIGURE 4:**
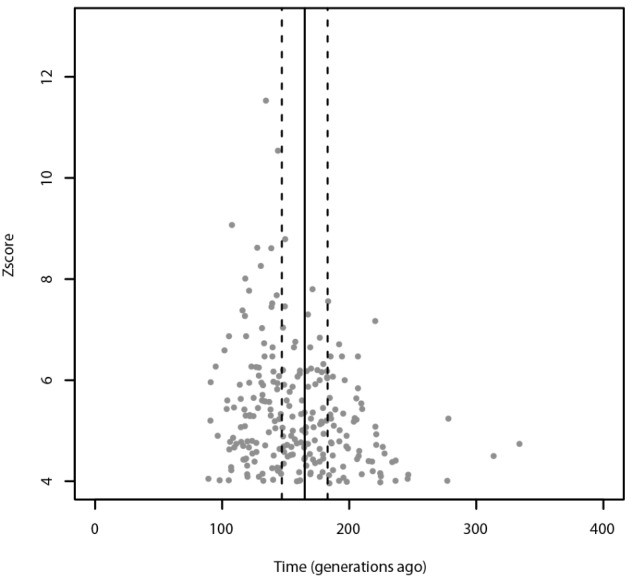
Admixture events identified with ALDER and MALDER. The gray dots represent significant admixture events detected with ALDER using Slovenians as target, the solid line represents the single admixture event detected using MALDER, dashed lines represent the confidence interval. Only the significant results after multiple testing correction are plotted. For ALDER results see Supplementary Table 5.

Using ALDER, the most significant admixture event was obtained with Russians and Sardinians as source populations and it happened 135 ± 9.31 generations ago (*Z*-score = 11.54). Moreover, we found significant signals also using Bedouin, and Russian population as possible sources of gene flow. When tested for multiple admixture events (MALDER), we obtained evidence for one admixture event 165.391 ± 17.1918 generations ago corresponding to ∼2620 BCE (CI: 3101–2139) considering a generation time of 28 years (Figure 4), with Kalmyk and Sardinians as sources. Most of admixture events involve Sardinians as one of the source populations coupled with North-East Europeans, Caucasian populations, and Western European populations (see Supplementary Table 1 for additional details). All significant results for ALDER are summarized in Supplementary Table 5.

We then modeled the Slovenian population as target of admixture of ancient individuals from Haak et al. (2015) while computing the f3(Ancient 1, Ancient 2, Slovenian) statistic. The most significant signal was obtained with Yamnaya and HungaryGamba_EN (*Z*-score = -10.66), followed by MA1 with LBK_EN (*Z*-score -9.7) and Yamnaya with Stuttgart (*Z*-score = -8.6) used as possible source populations (Supplementary Figure 5). Overall, we found a significant signal when we used Yamnaya and Early Neolithic genomes as potential admixture sources. Using ALDER, we modeled the time of admixture using Yamnaya with LBK_EN and Yamnaya with HungaryGamba_EN as possible ancient sources of admixture. We found a significant signal of admixture by using both pairs as ancient sources. Specifically, for the pair Yamnaya and Hungary_EN the admixture event is dated at 134.38 ± 23.69 generations ago (*Z*-score = 5.26, *p*-value of 1.5e-07) while for Yamnaya and LBK_EN at 153.65 ± 22.19 generations ago (*Z*-score = 6.92, *p*-value 4.4e-12). Outgroup f3 with Yamnaya put Slovenian population close to Hungarians, Czechs, and English, indicating a similar shared drift between these population with the Steppe populations (Supplementary Figure 6).

### Signals of Selection in Candidate Variants

Considering the pattern of admixture in Slovenian population, we searched for highly differentiated loci between Slovenians and specific reference populations. By comparing Russians and Slovenians we found that rs4129267 on *IL6R* is significantly differentiated with increase derived allele frequency in Slovenians. This marker is associated with several traits including C-reactive protein, fibrinogen and protein quantitative traits. Moreover, we found that rs603424 on *PKD2L1* shows higher derived allele frequency in Slovenians compared to Russians: this marker is significantly associated with metabolic traits and glycerophospholipid levels. Furthermore, the pattern of derived allele frequency is highly negatively correlated with latitude in our dataset (Pearson = -0.88, *p*-value = 6.38e-16). On the other hand, PCAdapt analysis using as reference South Europeans and Sardinians reveals significant differentiation for pigmentation allele rs12913832 (linked to blue eye color) in the *HERC2* gene. The derived allele frequency of rs12913832 in Slovenians and Russians is 0.64 and 0.86, respectively, which is higher when compared to Greeks and Spanish showing a derived allele frequency of 0.25 and 0.29, respectively. Moreover, we found a significant differentiation for three polymorphisms (rs1535, rs174547, and rs174550) in *FADS1* and *FADS2* genes which are implicated in lipid traits showing higher derived allele frequency in Greeks when compared to the Slovenians. Interestingly, we identified a signal on *BACE2* for rs6517656: this polymorphism shows a high derived allele frequency in Europe, Middle East and Asia while the ancestral allele is still predominant within Africa. A graphical representation of the worldwide allele variation is shown in Supplementary Figure 7 and the characteristics of selected GWAS SNPs are reported in Table 1 and Supplementary Table 6 with their frequency in each population in Supplementary Table 7.

**Table 1:** GWAS-SNP with FDR < 0.1 in selection scan.

Chr	Position (bp)	Gene	SNP	Comparison
1	154453788	IL6R	rs4129267	SLO vs. RUS
7	99642556	ZSCAN25	rs10242455	SLO vs. GRE
10	100315722	PKD2L1	rs603424	SLO vs. RUS
11	61803311	FADS1	rs174547	SLO vs. GRE
11	61804006	FADS1	rs174550	SLO vs. GRE
11	61830500	FADS2	rs1535	SLO vs. GRE
14	92460608	SLC24A4	rs10498633	SLO vs. ITN
15	28120472	HERC2	rs12913832	SLO vs. GRE
				SLO vs. SAR
				SLO vs. SPA
16	54455881	LOC105371272/73	rs2388639	SLO vs. ITN
19	32873722	SLC7A9 – CEP89	rs8101881	SLO vs. ITN
21	41211811	BACE2	rs6517656	SLO vs. SPA
				SLO vs. ITN


*For population acronyms see Supplementary Table S1.*

## Discussion

Slovenia presents a peculiar landscape, composed by mountain regions on the North West changing to flat lands toward the East. The border with the West not only represents a geographical barrier but also a linguistic one. To date no previous studies have described the genetic variation in Slovenian population. We considered different measurements of population admixture, isolation, and selection. In this study, we addressed the genetic features of Slovenian genomes and how admixture and selection shaped the genetic diversity of this population. Analysis of Y chromosome variation showed a presence of two main haplogroups R1b and R1a, confirming previous findings and suggesting gene flow from the Steppe (Haak et al., 2015). According to Zupan et al. (2013) analyses on Y chromosome variation suggested the existence of a common ancestral Slavic population in central European region. This would be in agreement with our findings highlighting a major contribution of genetic diversity from the Steppe.

From autosomal data our analyses on population structure revealed the absence of strong substructure within the Slovenian samples, although the samples came from different regions of the country. We discovered a strong affinity between Slovenians and Central-Eastern European populations such as Czechs and Hungarians. Slovenians are closer to North European samples respect to South European ones including the neighboring North Italian population. Our findings suggest that the Slovenian ancestry seems more closely related to population from Northern-Central Europe, respect to Western-South Europe.

For purpose of further studies focused on genetic epidemiology, our analyses show that Slovenians have no evidences of isolation. Nevertheless, we found a specific pattern of ROH hotspot in our Slovenian samples, despite the limitation of the possibility to replicate this pattern in other populations of the dataset due to the difference of sample size. Some of these regions contains interesting genes linked to GWAS traits including Type 1 Diabetes that should be further investigated.

Our analyses of admixture events using methods based on LD decay revealed that the modern Slovenian gene pool could be explained by several admixture events that happened in a single window of time. We also showed that that there is no support for multiple admixture events across time. Overall, the Slovenian genetic pool seems to have been formed during the Bronze Age period as admixture between North-Eastern European populations and Near-Eastern populations as proxy. This pattern has been further confirmed when we used ancient genomes. Specifically, we obtained the strongest signal for Slovenians using as references Yamnaya and Hungary Early Neolithic samples. The estimated admixture time falls within the range of that one obtained using modern populations. We can conclude that populations closely related to Yamnaya and early Neolithic Hungarians contributed during the Bronze age to the foundation of the modern genetic variability in Slovenians. We could make the hypothesis that also disease and specific traits-alleles were likely to have been introduced in the Slovenian genetic pool during this period, such as pigmentation alleles (including the high frequency of blue eyes alleles found in Slovenian samples), lactose tolerance (rs4988235, whose frequency in Slovenia is 0.36) and also immune related alleles such as rs4833095 in TLR1 (Slovenian derived allele frequency of 0.7, Bronze Age ∼0.8).

Considering the discovered admixture pattern that contribute to the actual genetic diversity of this populations, we analyzed the possible selection signals in a broader context. We specifically focused on putatively selected variants in this population that could be useful for genetic epidemiology and to better understanding the forces shaping the genetic diversity in the Slovenians. Our selection study revealed significant hits on markers associated mainly on lipid traits and eye pigmentation when compared to South Europeans (Greeks) such as *HERC2* for blue eye color and *FADS1-FADS2* alleles; *FADS* genes are essential for the synthesis of long-chain unsaturated fatty acids (Teslovich et al., 2010). These genes have been previously found under selection in different populations from East Asia (Auton et al., 2015) and Greenland (Fumagalli et al., 2015), with also evidence of ancient selection in Africa (Ameur et al., 2012). Finally, in Mathieson et al. (2015) the *FADS1* and *FADS2* loci were found under selection in Neolithic samples from Europe. This pattern suggests that these genes played an important role in human adaptation to different diets and dietary habits in Slovenian ancestors could have played a role in the selection pressure for these genes.

On the other hand, when we compared Slovenians with North-Eastern populations, such as Russians, Slovenians showed signature of selection in *PKD2L1* which is a gene involved in taste (Fujikura, 2016) and also possibly in olfaction (Keydar et al., 2013) and therefore could be also linked to dietary patterns in Slovenians. We also found that the frequency of the putatively selected allele shows high negative correlation with latitude and its derived frequency in Slovenians is very similar to North Italians, whereas populations as Czechs and Lithuanians show much lower derived allele frequency. This further suggests a putative link with different dietary habits associated with latitude that could have affected the variation in *PKD2L1*. Another signal has been found on *IL6R*: this gene was discovered as underlying coronary artery disease (Byars et al., 2017) and harboring signature of selection and presence of antagonistic pleiotropic trade-offs. Moreover, *IL6R* was discovered to suppress feeding and improves glucose tolerance in mice (Timper et al., 2017). Overall, using our approach we found several genes linked to diet that could explain possible selection forces on these alleles that were introduced previously from different sources in the Slovenian genetic pool. Our results show that the pattern of selected genes in modern Slovenians was shaped by several admixture events during the Bronze Age. Slovenian population shows that some GWAS specific alleles differentiated with both North-Eastern European population and South European populations.

One limitation of our study is the use of only SNP-chip data, future studies involving whole-genome sequencing would highlight more details the genetic features of the genes under selection, also enhancing the power to detect putatively deleterious rare variants.

## Author Contributions

MM and PM designed the project, performed the analyses, and wrote the manuscript. MR-G and DG collected the samples. PG, MR-G participated in project coordination and helped to draft the manuscript. All authors read and approved the final manuscript.

## Conflict of Interest Statement

The authors declare that the research was conducted in the absence of any commercial or financial relationships that could be construed as a potential conflict of interest.
