# Supplementary material for: Genetic Landscape of Slovenians: Past Admixture and Natural Selection Pattern
Source: Front Genet. 2018 Nov 19;9:551. doi: 10.3389/fgene.2018.00551 (PMC6252347; doi:10.3389/fgene.2018.00551)
Supplement: Supplementary file 2 [file Data_Sheet_2.PDF]

Supplementary Figure 1: a) PCA including Slovenian samples only. Individuals are coloured according to the region of origin and there is no evidence for population structure within Slovenia. See Figure 1 for details of sampling locations. b) PCA of Slovenian samples with European populations (PC2 vs PC3).

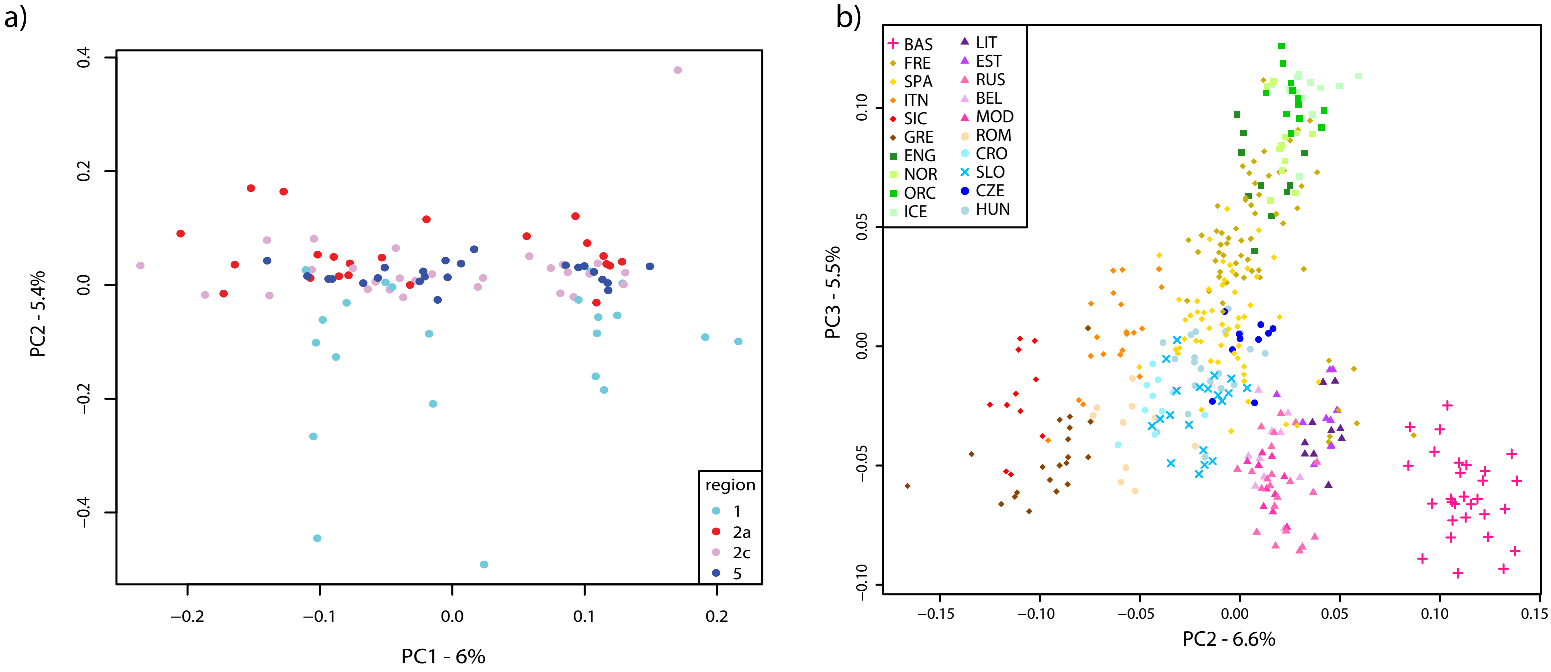

Supplementary Figure 2: PCA including Slovenian samples and populations worldwide distributed (127,385 markers). This analysis is based on the Slovenian_HO dataset and the list of populations is reported in Supplementary Table 1.

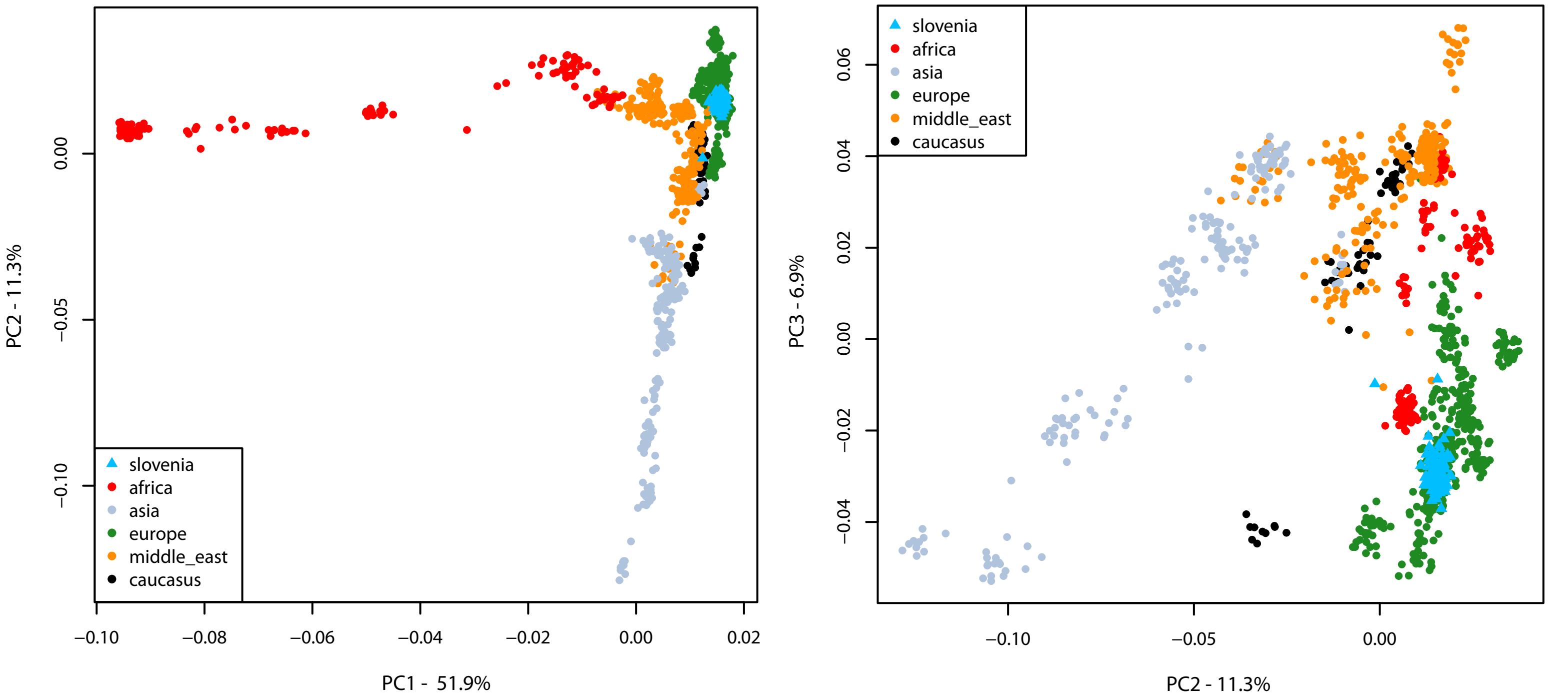

Supplementary Figure 3: Choice of the most likely number of clusters. The analysis shows that the most probable number of cluster is k=6 supported by different models implemented in Mclust. BIC: Bayesian Information Criterion. The number of components showing the overall lowest BIC value is k=6. The name of the models are sepcified with a three letter acronym describing volume, shape and orientation of the distribution. The acronyms reported in the figure legend stand for: EII = spherical, equal volume; VII = spherical, unequal volume; EEI = diagonal, equal volume and shape; VEI = diagonal, varying volume, equal shape EVI = diagonal, equal volume, varying shape; VVI = diagonal, varying volume and shape; EEE = ellipsoidal, equal volume, shape, and orientation; EVE = ellipsoidal, equal volume and orientation; VEE = ellipsoidal, equal shape and orientation; VVE = ellipsoidal, equal orientation; EEV = ellipsoidal, equal volume and equal shape; VEV = ellipsoidal, equal shape; EVV = ellipsoidal, equal volume; VVV = ellipsoidal, varying volume, shape, and orientation .

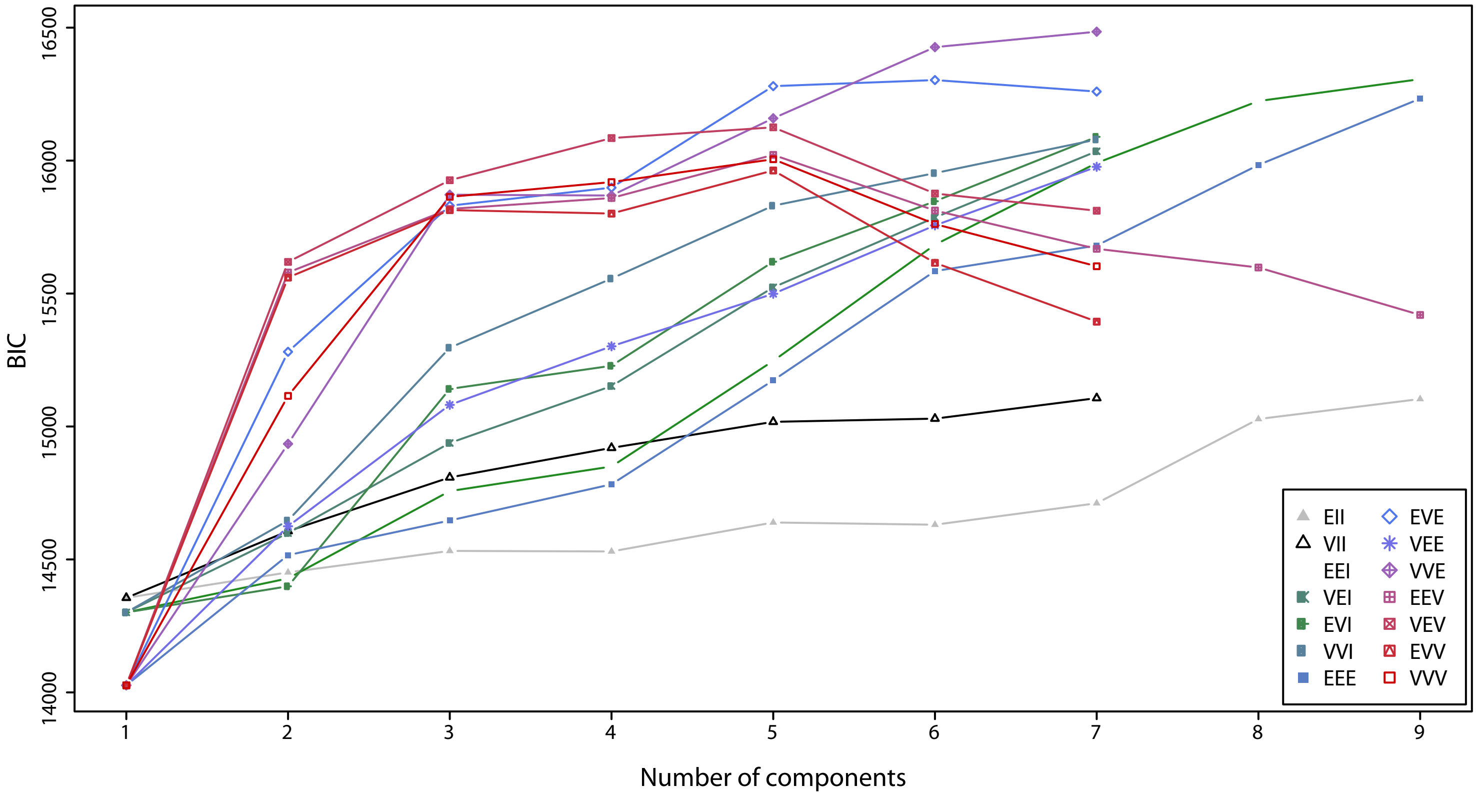

Supplementary Figure 4: UPGMA tree based on Fst matrix. The red arrow indicates the position of the Slovenian samples. All populations listed in Supplementary Table 1 were included without any filter for sample size

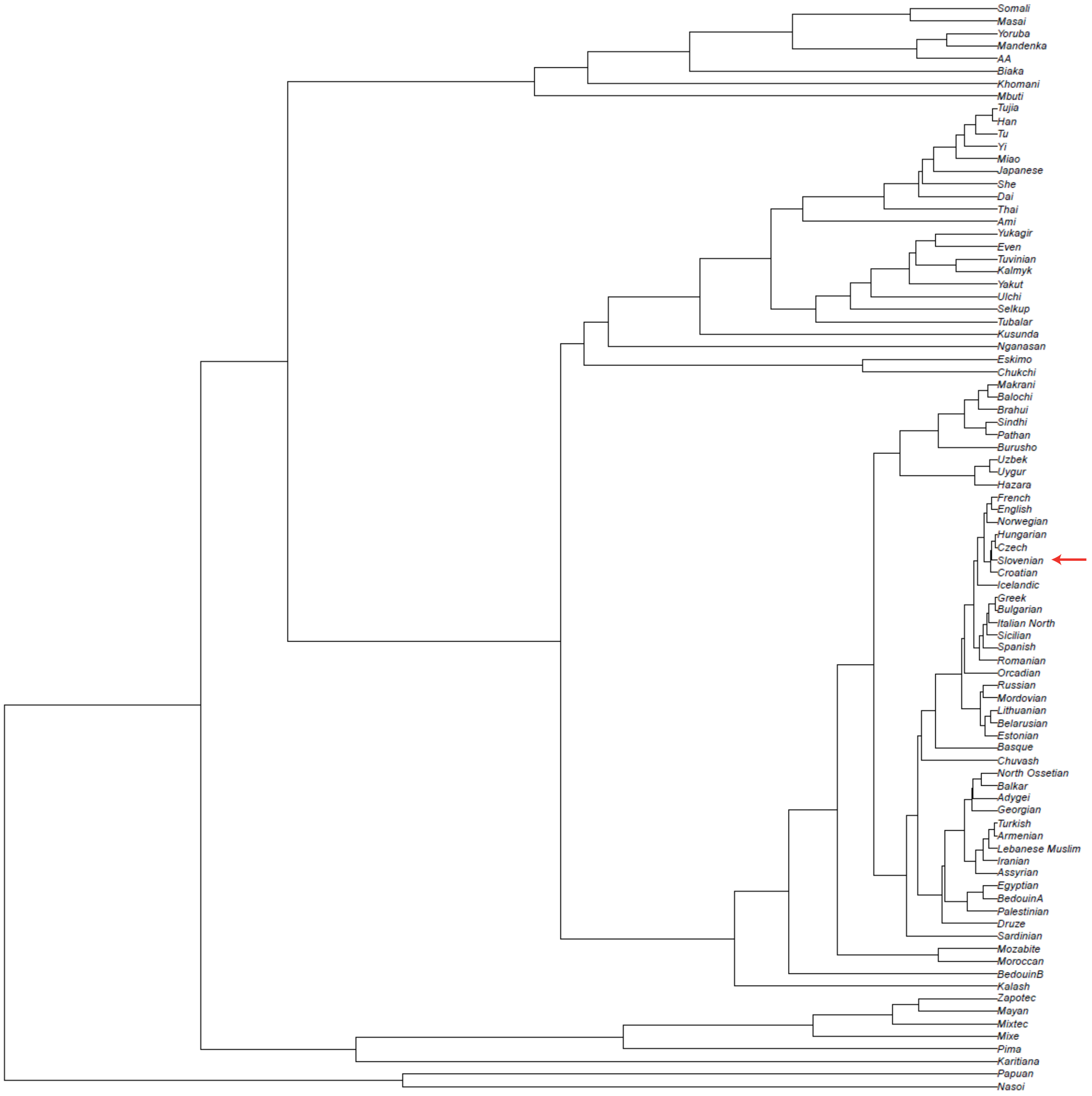

Supplementary Figure 5: Heatmap of admixture signals using all different combination of ancient genomes. We tested all combinations f3(Ancient1, Ancient2, Slovenian). The target of the admixture event is always represented by the Slovenian population while all possible pairs of ancient genomes have been used as source populations. In the heatmap we plotted the Z-score for each f3 analysis and the red colour represents f3 values with Z-score < -3, which we interpret as significant. For sample acronomys see Supplementary Table 1.

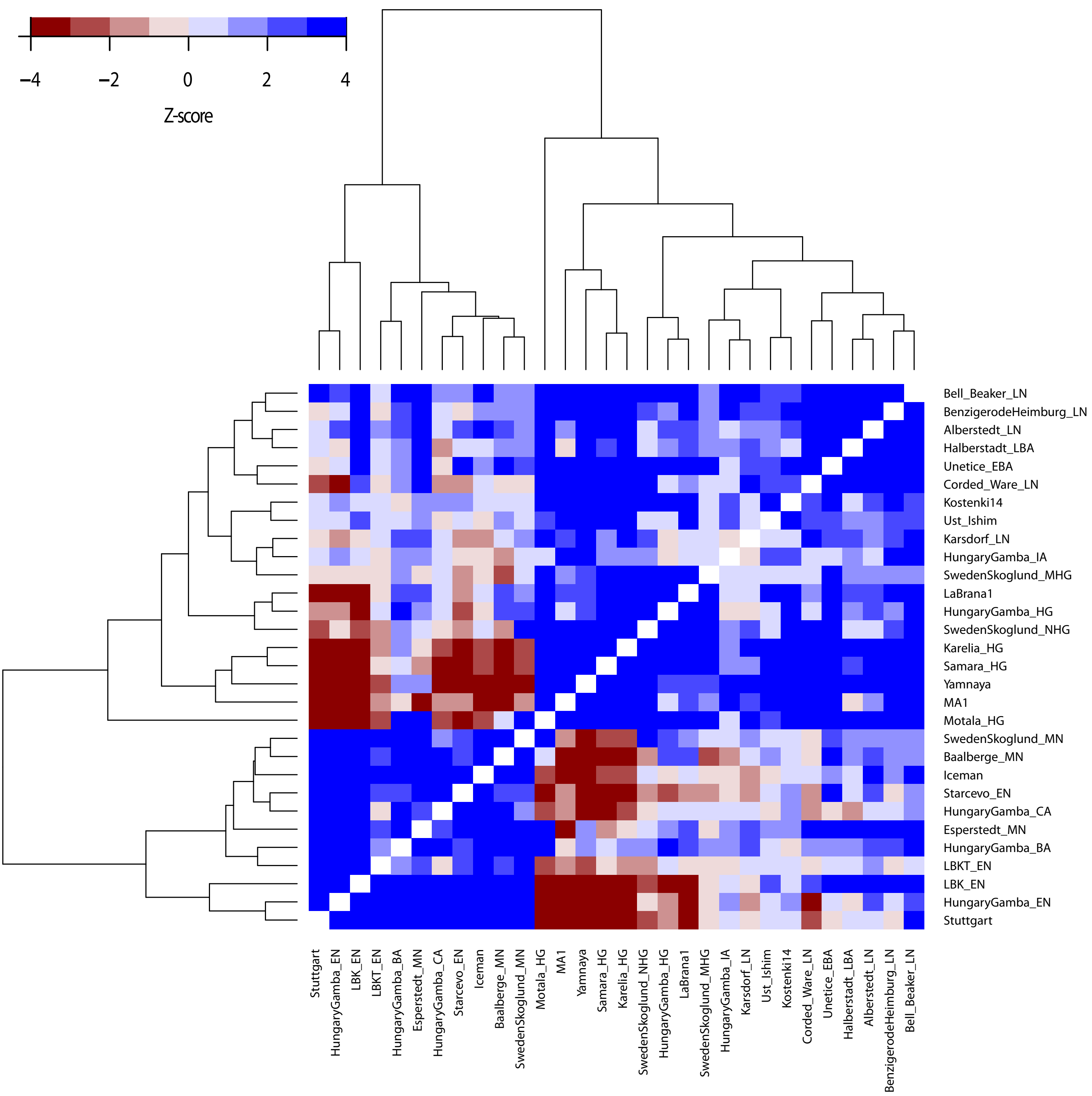

Supplementary Figure 6: Outgroup f3 statistic for Yamnaya in our dataset. We used Yamnaya as a source population to check the genetic contribution of steppe diversity in each of our population in the dataset. Slovenian samples (represented by the red square) are placed close to English and Croatian samples. For population acronyms see Supplementary Table S1.

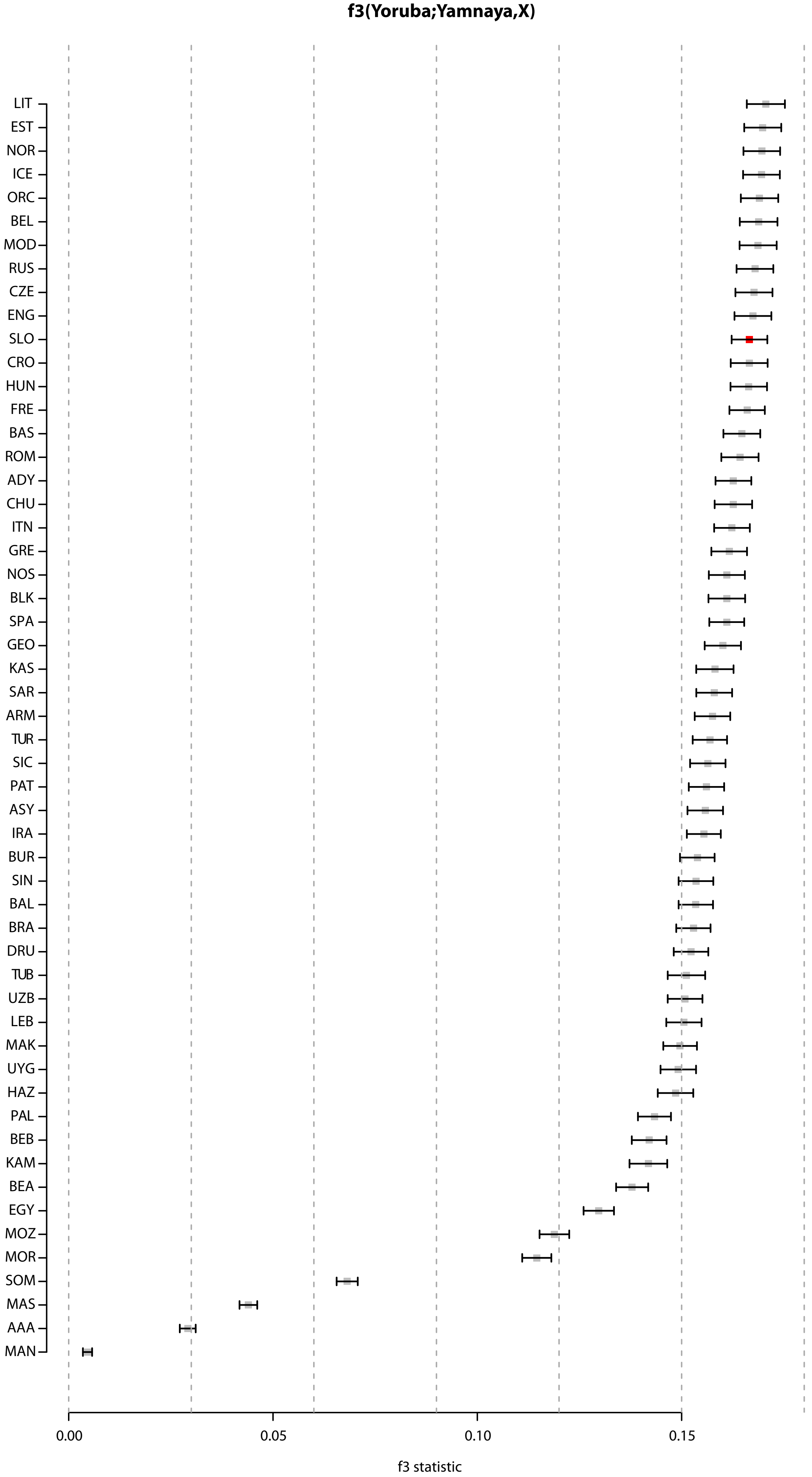

Supplementary Figure 7: Piechart of allele frequency of significant markers found in PCadapt analyses. Piecharts with white borders represent significat comparisons. A: ancestral; D: derived. For population acronyms see Supplementary Table S1.

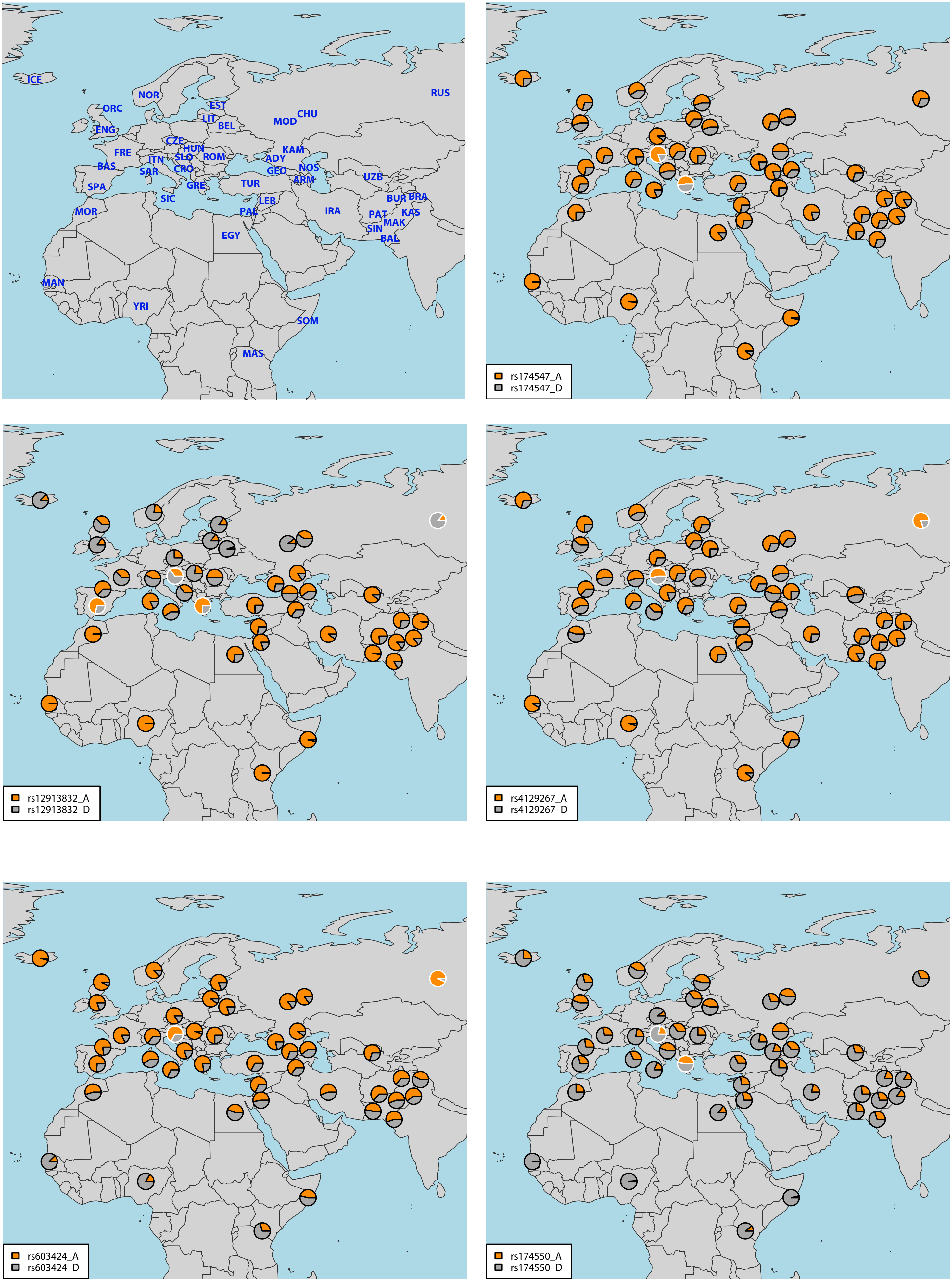

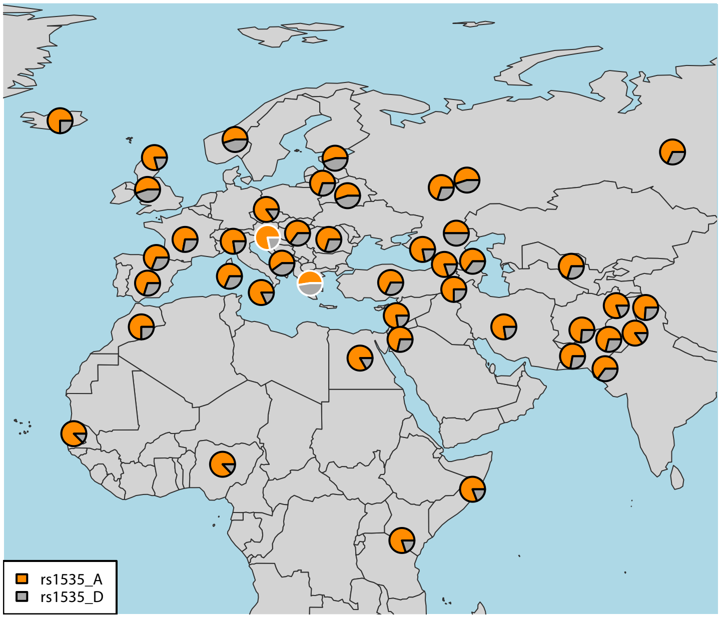
rs1535_A
rs1535_D

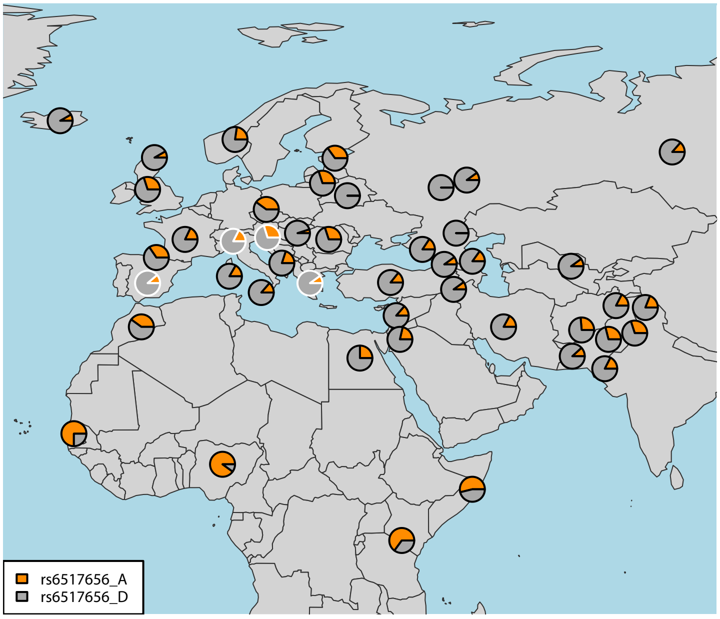
rs6517656_A
rs6517656_D
